# Exercise Affects the Formation and Recovery of Alcoholic Liver Disease through the IL-6–p47^phox^ Oxidative–Stress Axis

**DOI:** 10.3390/cells11081305

**Published:** 2022-04-12

**Authors:** Wei Cui, Xiang Li, Weiyue Xue, Huiting Wei, Gang Zhou, Ye Qiu, Di Cui

**Affiliations:** 1 Department of Physical Education, Hunan University, Changsha 410000, China; cuiwei@hnu.edu.cn (W.C.); LiXiang0824@hnu.edu.cn (X.L.); s212301567@hnu.edu.cn (W.X.); w1486776921@hnu.edu.cn (H.W.); zg460@hnu.edu.cn (G.Z.); 2 College of Biology, Hunan University, Changsha 410000, China; qiuye@hnu.edu.cn

**Keywords:** alcoholic liver disease (ALD), NADPH oxidase (NOX), interleukin-6 (IL-6), oxidative stress, hepatocellular, inflammation, exercise

## Abstract

(1) Background: To explore the effect of exercise on the formation and recovery of alcoholic liver disease (ALD) and whether the IL-6–p47^phox^ oxidative–stress axis is involved in that process. (2) Methods: Firstly, 23 six-week-old male C57BL/6J mice were randomly divided into the Con group, ALD group, ALD + NOXI group, ALD + Ex group, and ALD + Ex + NOXI group. The Liber–DeCarli alcoholic liquid diet was used for 6 weeks to establish the ALD mice model, and the Con group was given the TP4030C control diet. The remaining groups were fed with the TP4030B alcoholic diet, and exercise intervention was started after the ALD model establishment and lasted for another 6 weeks, with or without administration of the NOX inhibitor apocynin by intraperitoneal injection on every exercise training day. Secondly, 28 mice were randomly divided into the Sed group, Eth group, Eth + Ex group and Eth + Ex + NOXI group. The Sed group was given the TP4030C control diet. The remaining groups were fed with the TP4030B alcoholic diet and exercise intervention was started synchronously combined with or without administration of intraperitoneal apocynin injections on every exercise training day for 5 weeks. After each individual experiment was accomplished, physiological assessment and biochemical analysis of blood and tissue samples were examined. (3) Results: The levels of TG in serum and IL-6 protein content in liver tissue in the ALD group were significantly increased compared to the Con group (*p* < 0.05); compared with ALD, p47^phox^ expression in muscle was increased significantly in the ALD + NOXI group (*p* < 0.05), and TG in serum decreased in the ALD + Ex group (*p* < 0.05). TG in serum, AST/ALT ratio, and IL-6 content in both liver and muscle decreased (*p* < 0.05) in the ALD + Ex + NOXI group with MDA in muscle significantly increased (*p* < 0.01). The AST/ALT ratio, TG in serum, SOD in liver, and p47^phox^ in both liver and muscle in the ALD + Ex + NOXI group were significantly decreased compared with the ALD + NOXI group (*p* < 0.01). Compared with the ALD + Ex group, the liver index and HDL-C levels in serum were decreased (*p* < 0.05) in the ALD + Ex + NOXI group. The degree of hepatocyte steatosis and inflammatory infiltration were ameliorated after exercise intervention. In the Eth group, the relative epididymal fat content, HDL-C level, and AST/ALT ratio were significantly decreased, and TG and gp91^phox^ in liver were significantly higher than in the Sed group (*p* < 0.05, *p* < 0.01). Compared with the Eth group, the AST/ALT ratio, MDA in the liver, and NOX4 and p47^phox^ protein expression in the liver were significantly increased, and body weight decreased significantly in the Eth + Ex group (*p* < 0.05, *p* < 0.01), as did TG in the liver and MDA in muscle. In the th + Ex + NOXI group, gp91^phox^ expression in the liver and body weight were significantly decreased (*p* < 0.05, *p* < 0.01). In the Eth + Ex + NOXI group, the ratio of AST/ALT and MDA in muscle were increased when compared with the Eth + Ex group, and the protein expression of gp91^phox^ and p47^phox^ were much lower (*p* < 0.01). (4) Conclusions: 6 weeks of exercise intervention during the recovery phase of ALD ameliorates hepatocyte damage and dyslipidemia through the IL-6–p47^phox^ oxidative–stress axis, and applying a NOX inhibitor in combination could optimize this. However, drinking alcohol during exercise exacerbates dyslipidemia and oxidative stress, with hepatocyte IL-6–p47^phox^ downregulated.

## 1. Introduction

Excessive drinking has become an important public health problem worldwide with the increase in alcohol consumption, drinking frequency, binge drinking, and the change in people’s diet structures. Studies on the etiology of alcoholic liver disease (ALD) at the cellular and molecular levels suggest that the pathogenesis may be attributed to hepatotoxic ethanol and its metabolites [1]. In addition, immune damage [2], oxidative stress response [3], inflammatory response of cytokines [4,5], endotoxin [6,7], heredity [8,9], and other factors have been proven implicated as well. Interleukin-6 (IL-6) plays a key role in the occurrence and development of liver diseases and the effects vary in different periods of disease progression [10]. At the early stage of ALD, high levels of IL-6 in serum suggest a good prognosis, and in hepatocytes, its downstream signal transducer and activator of transcription 3 (STAT3) activation ensues to regulate various hepatocyte-protective genes to reduce inflammatory response; however, high levels of serum IL-6 serve as a poor prognostic indicator for patients at the terminal stage of ALD [11,12]. Therefore, targeting IL-6 and its related signaling pathways in the liver could hint at a new target for the diagnosis and treatment of liver disease. Recently, microRNA-223 was reported to ameliorate ALD symptoms by inhibiting the neutrophil IL-6-p47^phox^ (NADPH oxidase p47^phox^) oxidative-stress pathway by Man et al., a research team from the American Institute on Alcohol Abuse, concluding that the phosphorylation of p47^phox^ was induced by stimulating hepatocytes with inflammatory cytokine IL-6 [13]. NADPH oxidase (NOX) is an enzyme family that catalyzes the production of superoxide from oxygen and its excess activation was confirmed to cause oxidative stress in the liver and to aggravate inflammatory response by producing a large number of reactive oxygen species in a very short period of time [14,15]. According to our preliminary data, the transcription and protein expression of NOX2 (gp91^phox^) and NOX4 were detected in stressed livers [16]. As a cytoplasmic subunit of both NOX2 and NOX4, p47^phox^ functioned under the conditions of inflammatory mediators and cytokines such as IL-6. Excessive alcohol consumption resulted in structural damage to hepatocytes and in the disruption of the tricarboxylic acid cycle, which activates Kupffer cells to induce phosphorylation and the nuclear translocation of transcription factors. At the same time, a large number of inflammatory mediators are released to activate signaling pathways. Appropriate physical exercise and ischemia both enhance biological defense mechanisms [17,18]. Regular exercise is beneficial for our health and can improve the physiological and pathological changes from ALD. [19,20] Exercise ameliorates the sensitivity of liver tissue to insulin, including the rapid regulation of blood glucose levels, and promotes liver glycogen decomposition and the hydrolysis of fat [21]. Exercise alleviates lipid peroxidation by improving the activities of lipolytic enzymes in muscle and liver, enhances energy utilization [22], and improves the activity of circulating fatty acids and insulin [23]. In addition, exercise-stressed skeletal muscle secretes IL-6, serving as a “cytokine”, to play an anti-inflammatory role by downregulating the levels of pro-inflammatory factors in other tissues and by inhibiting the activation of pro-inflammatory mediators so as to exert anti-inflammatory functions [24]. To date, the role of IL-6 is still undefined in ALD pathology derived from hepatocytes or skeletal muscle, and the effect of exercise on the IL-6–p47^phox^ oxidative–stress axis during the ALD process and recovery needs to be further elucidated. In the present research, we explored the effects of aerobic exercise and a NOX inhibitor, apocynin, on both liver and skeletal muscle tissue after the recovery and during the formation of ALD, aiming to unveil the internal molecular mechanism of the ALD amelioration induced by exercise and to pave a novel route for ALD treatment and prevention.

## 2. Materials and Methods

### 2.1. Animal Grouping and Model Preparation

The experimental subjects were male C57BL/6J mice, 6 weeks old, body weight (18.95 0.38) g, purchased from Hunan SJA Laboratory Animal Co., LTD, Hunan, China, animal license number: SCXK:2019-0004. The experimental scheme was approved by the Animal Experiment Ethics Committee of Hunan University (Acceptance number: HNUBI0202101007). Feeding conditions: natural day and night light, ambient temperature (24 ± 2), relative humidity 55–60%, and good ventilation.

In the first experiment, 23 mice were randomly divided into 5 groups, Con group (*n* = 5), ALD group (*n* = 4), ALD + NOXI group (*n* = 4), ALD + Ex group (*n* = 5), and ALD + Ex + NOXI group (*n* = 5). The ALD model was established by feeding with Lieber–DeCarli liquid diet for 6 weeks as recommended in the protocol by KJ Thompson [25] and Adeline Bertola [26]. Briefly, mice were first fed with TP4030C control liquid diet for 3 days accommodation; then, mice in Con group were still fed with TP4030C control liquid diet till the end, while mice in other groups were fed with TP4030B (high-fat with 5.07% alcohol) and TP4030C mixture diet in the ratios of 1:4, 2:3, 3:2 and 4:1, respectively, for 4 days accommodation, followed by feeding with TP4030B high-fat alcoholic liquid diet for the remaining 5 weeks, and the total ALD model establishing period lasted 6 weeks. After modeling, the diet of all mice were switched from Liber–DeCarli liquid diet to free normal solid chow and water. Mice with exercise intervention (groupings labeled with Ex) underwent a swimming aerobic training program, specifically including a pre-swim for 10 min the first day, with a gradual increase of 10 min each day until a total time of 1 h to sustain the level at 1 h/day, 6 days/week for another 6 weeks. By contrast, the other mice were kept sedentary. Mice with drug intervention (groupings labeled with NOXI) were intraperitoneally injected with NOX inhibitor apocynin at 10 mg/kg on each exercise training day, with the same amount of placebo for the remaining mice.

In the secondary experiment, 28 mice were randomly divided into 4 groups, Sed group (*n* = 6), Eth group (*n* = 6), Eth + Ex group (*n* = 4), and Eth + Ex + NOXI group (*n* = 5). The exercise intervention protocol was following the first experiment instruction for mice grouping labeled with Ex. Totally different from experiment one, the alcohol dietary intervention was executed synergistically with exercise training. Specifically, mice groupings labeled with Eth were fed with TP4030B high-fat alcoholic liquid diet from the secondary week according to the ALD modeling protocol in experiment one, while mice in Sed group were still fed with TP4030C control liquid diet, and the total experiment consumption time was 6 weeks. The NOX inhibitor apocynin was injected following the same protocol of experiment one for mice groupings labeled with NOXI, and the remaining groups with the same amount of placebo.

### 2.2. Feed and Reagents

Solid normal chow was purchased from Hunan SJA Laboratory Animal Co., LTD. Liber–DeCarli liquid feed (TP4030C and TP4030B) was purchased from Trophic Animal Feed High-tech Co., LTD, Jiangsu, China, and the TP4030C (1 kcal/mL) energy distribution was composed of 18% fat, 47% protein, and 35% other carbohydrates, while TP4030B comprised 35% fat, 28% alcohol, 18% protein, and 19% other carbohydrates.

Apocynin (A606538-0025) was purchased from Sangon Biotech (Shanghai, China). Alanine transaminase (ALT), aspartate transaminase (AST), malondialdehyde (MDA), triglycerides (TG), low-density lipoprotein cholesterol (LDL-C), high-density lipoprotein cholesterol (HDL-C) kits were purchased from Nanjing Jiancheng Bioengineering Institute (Nanjing, China). NOX4 (SC-518092), gp91^phox^ (SC-130543), p47^phox^ (SC-17844), and IL-6 (SC-57315) primary antibodies were purchased from Santa Cruz (Shanghai, China). β-actin (BS6007MH) primary antibody was purchased from Xianzhi Biotechnology Co., LTD (Hangzhou, China). HRP-labeled secondary antibody (BST13IO7A50) was purchased from Boster Biological Technology Co., LTD (Wuhan, Chian).

### 2.3. Mice Keeping and Harvesting

All animals’ general behaviors and living conditions were observed and recorded daily, including appetite, hair, mental state, urine, feces, etc. The mice’s body weights were measured once a week. Before sacrifice, a glucose tolerance test was conducted after 16 h’s fasting. The whole liver, epididymis fat, bilateral gastrocnemius muscles, and serum were obtained for future detection after the mice been euthanized under anesthetic.

### 2.4. Glucose Tolerance Testing

Mice were given 20% glucose solution to detect a change in blood glucose with a Roche blood glucose meter, with 10 mg/mL intragastrically or intraperitoneally administered at 0, 30, 60, 120 min after glucose administration.

### 2.5. Spectrophotometric Density Value Analysis

Liver and skeletal muscle tissue stored at −80 °C were homogenized by Phosphate Buffer Saline to prepare samples for examination. Commercialized kits from Nanjing Jiancheng Institute of Biological Engineering (Nanjing, China) were used to detect serum ALT, AST, TG, LDL-C, HDL-C, and TG in liver, according to the instructions. The level of MDA was determined by the thiobarbituric acid method, and the activity of superoxide dismutase (SOD) was determined by pyrogallol. Chemical colorimetry was applied to measure the optical density values.

### 2.6. HE and Oil Red O Staining

Paraffinized sections of liver tissues were successively dewaxed, hydrated, stained with hematoxylin, differentiated, washed with water, stained with eosin, dehydrated, made transparent, and sealed. The sampling and fixation procedures of Oil Red O were consistent with HE staining but used frozen sections to cut. The sliced tissue sections were 10μm. The stained slides were observed under light microscope for tissue steatosis, vacuoles, structural disorder, cell-like necrosis and inflammatory infiltration.

### 2.7. Western Blotting

Tissues were homogenized using a protein extraction RIPA buffer, with proteinase and phosphatase inhibitors (Beyotime Biotechnology, Shanghai, China), and the sample protein concentration was evaluated by BCA protein assay. Electrophoresis was performed on a 10–12% sodium dodecyl sulfate polyacrylamide gel, and then transferred to a PVDF membrane (Merck, Shanghai, China). The membranes were incubated with 5% non-fat milk in TRIS-buffered saline (pH 7.4) containing 0.1% Tween (TBST) for 2 h blotting. After washing several times with TBST buffer, the membranes were incubated in blocking solution with primary antibodies, including NOX4, gp91^phox^, p47^phox^ and IL-6 (antibody dilution rate of NOX4, gp91^phox^ and p47^phox^ were 1:500, IL-6 was 1:200, and β-actin 1:10,000) overnight at 4 °C Then, the goat anti-rat IgG with HRP-conjugated (1:5000) secondary antibody was applied for primary–secondary antibody reaction. Finally, enhanced chemiluminescence (ECL) was facilitated to detect protein expression. The densities of all bands on membranes were captured and quantified by using Image J software.

### 2.8. Statistical Analysis

Data are presented as the mean ± standard error (SE). Analysis was performed by GraphPad Prism version 6.01′s one-way ANOVA or *t* test, with statistical significance set at *p* < 0.05 and *p* < 0.01.

## 3. Results

### 3.1. Exercise Training Ameliorates Dyslipidemia and Inflammation

**Figure 1:**
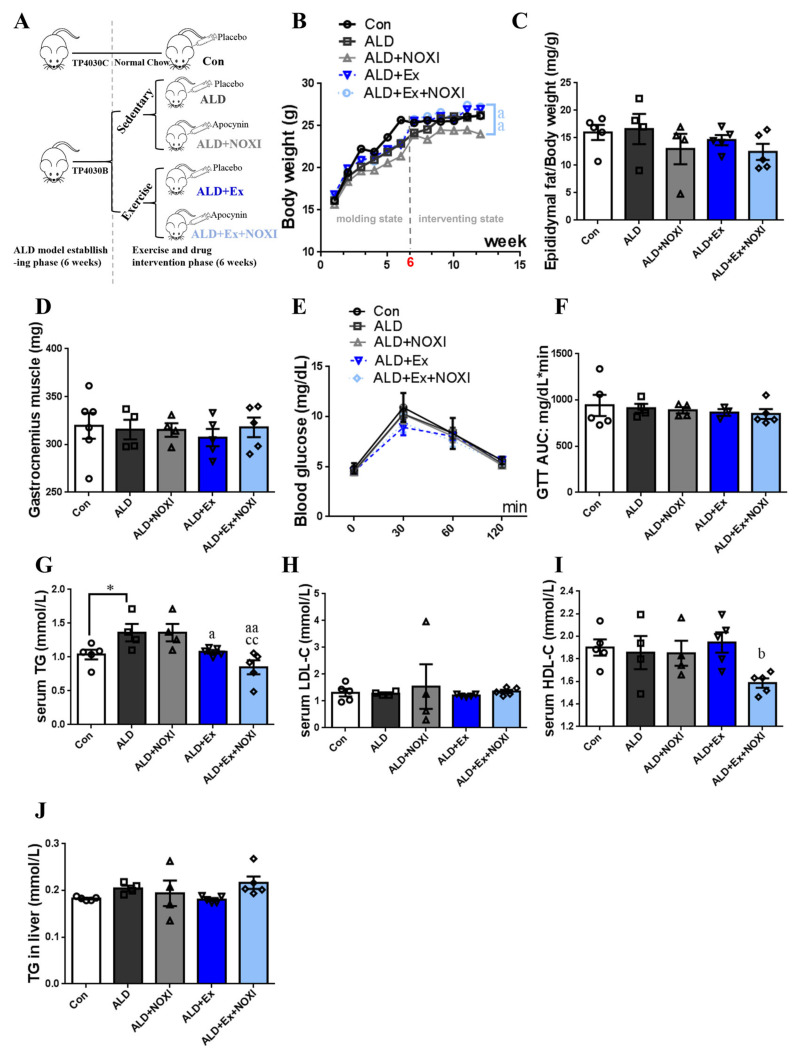
Inhibition of NOX affects the normal weight gain of mice, and exercise training ameliorates dyslipidemia and hepatocyte inflammatory damage. (**A**) Grouping of experimental animals. (**B**) Changes in body weight over 12 weeks. (**C**) Ratio of epididymal fat to body weight. (**D**) Gastrocnemius muscle weight. (**E**,**F**) Glucose change and area-under-the-curve in glucose tolerance test. (**G**,**J**) TG content in serum and liver tissue. (**H**) LDL-C in serum. (**I**) HDL-C in serum. (**A**–**J**) Con group, *n* = 5; ALD group, *n* = 4; ALD + NOXI group, *n* = 4; ALD + Ex group, *n* = 5; ALD + Ex + NOXI group, *n* = 5. The molding effect is represented by *, * *p* < 0.05. The exercise effect is denoted by a, ^a^ *p* < 0.05 and ^aa^ *p* < 0.01. The drug effect is denoted by b, ^b^ *p* < 0.05. The effect of combined exercise and drug intervention is indicated by c, ^cc^ *p* < 0.01.

The main physiological indexes and lipid metabolism levels of ALD mice under exercise and drug intervention are shown in Figure 1. The ALD model in mice was established 6 weeks before the experiment, and intervention was performed within 6 weeks of the recovery period after modeling (Figure 1A). The ALD + Ex + NOXI group gained weight more significantly than the ALD + NOXI group *(p* < 0.01, Figure 1B). Reaching the peak of blood glucose at about 30 min and returning to normal at about 120 min in each group, the range of blood glucose changes was small in mice after exercise intervention. (Figure 1E). Compared with the Con group, the level of TG in serum was increased significantly in the ALD group (*p* < 0.05). After exercise and drug intervention, serum TG levels of mice decreased significantly (*p* < 0.05, *p* < 0.01), but there were no significant differences in TG levels in liver tissues (Figure 1G,J). The levels of HDL-C in the ALD + Ex + NOXI group were decreased more significantly than in the ALD + Ex group (*p* < 0.05, Figure 1I).

### 3.2. Exercise Reduces Oxidative Stress Levels in Liver Tissue

**Figure 2:**
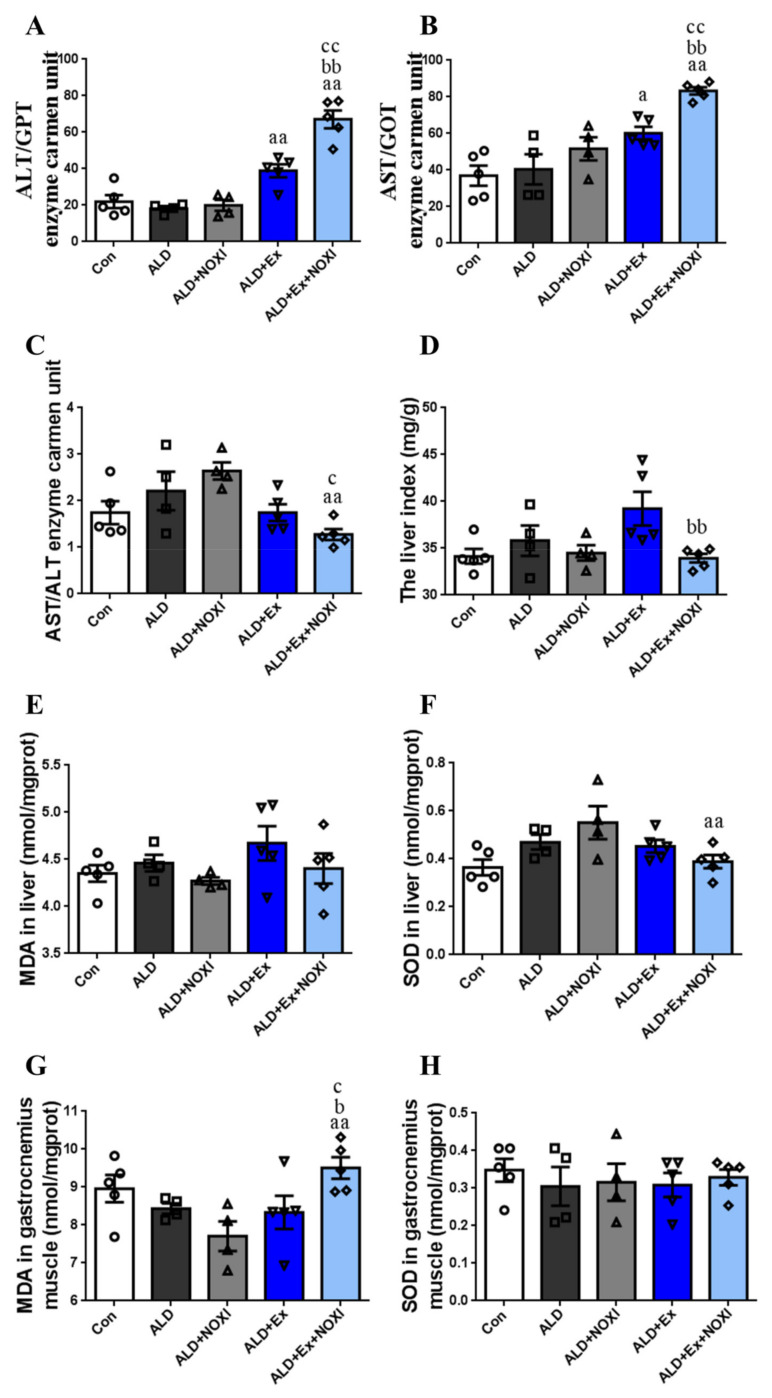
With the combined intervention of apocynin and exercise, oxidative stress levels were reduced in liver tissue and increased in gastrocnemius muscle tissue. (**A**,**B**) ALT and AST levels in serum. (**C**) Serum AST/ALT values. (**D**) The level index. (**E**,**F**) MDA and SOD levels in liver tissue. (**G**,**H**) MDA and SOD levels in gastrocnemius muscle tissue. The exercise effect is denoted by a, ^a^ *p* < 0.05, ^aa^ *p* < 0.01. The drug effect is denoted by b, ^b^ *p* < 0.05, ^bb^ *p* < 0.01. The effect of combined exercise and drug intervention is indicated by c, ^c^ *p* < 0.05, ^cc^ *p* < 0.01.

The effects of exercise and drug intervention on liver injury and oxidative stress of liver and gastrocnemius muscle tissue in ALD mice are shown in Figure 2. Compared with the ALD group, ALT and AST levels in the ALD + Ex group and the ALD + Ex + NOXI group were increased, and in the ALD + Ex + NOXI group, the levels of ALT and AST were increased significantly compared with those in the ALD + NOXI group and the ALD + Ex group (*p* < 0.01, Figure 2A,B). Compared with the ALD group and ALD + NOXI group, the AST/ALT values in the ALD + Ex + NOXI group were decreased significantly (*p* < 0.05, *p* < 0.01, Figure 2C). Exercise stress caused organ hypertrophy in mice liver, and the liver index of the ALD + Ex + NOXI group was significantly decreased after apocynin intervention (Figure 2D). Compared with the ALD + NOXI group, SOD levels in liver tissue were decreased after exercise intervention (*p* < 0.01, Figure 2F). In the ALD + Ex + NOXI group, the level of MDA in gastrocnemius muscle tissue was increased more than that in the ALD and ALD + Ex groups (*p* < 0.05), and also significantly more than that in the ALD + NOXI group (*p* < 0.01, Figure 2G).

### 3.3. Exercise Significantly Improved Fat Accumulation and Inflammatory Injury in Liver Tissue, and Its Positive Effect Is Involved with the Liver IL-6–p47^phox^ Axis

**Figure 3:**
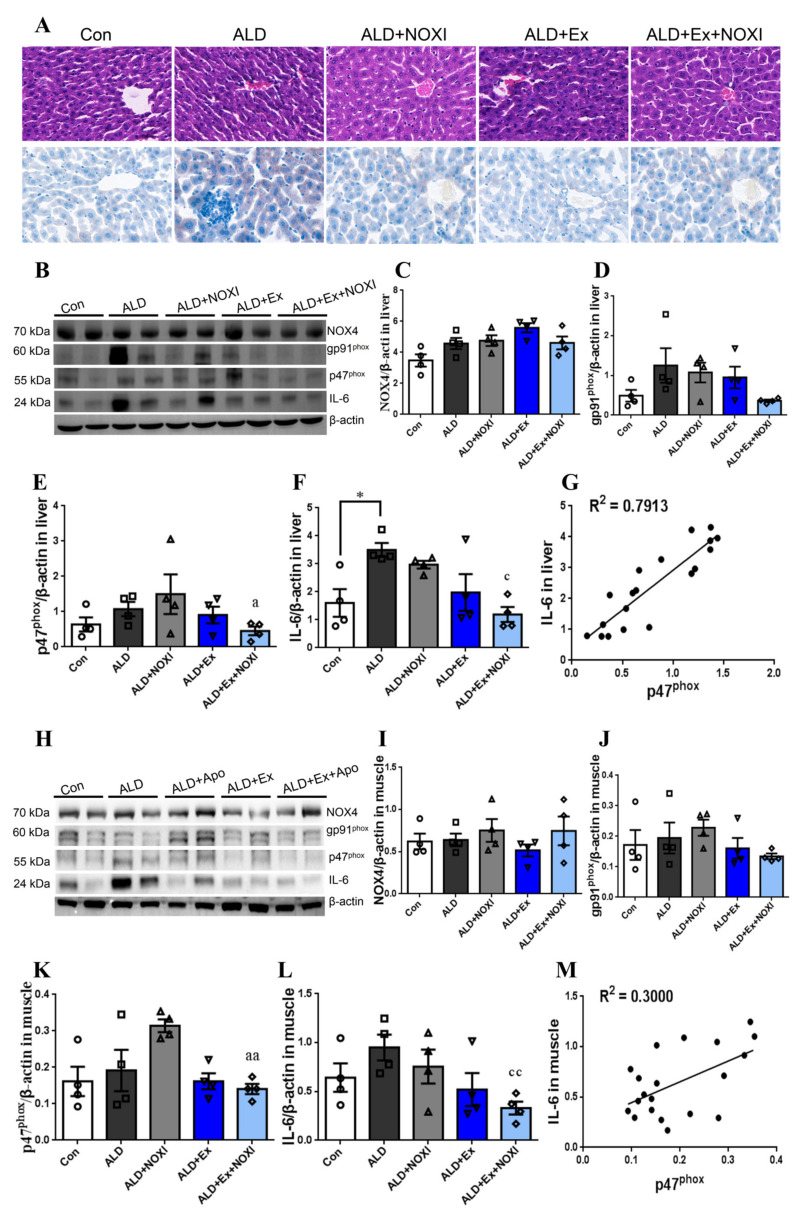
Exercise significantly improved fat accumulation and inflammatory injury in liver tissue. Exercise inhibited the expression of p47^phox^ and IL-6 proteins, and they were highly correlated in liver tissue. (**A**) The upper row is the result of HE staining, and the lower row is the result of Oil Red O staining. (**B**–**F**) NOX4, gp91^phox^, p47^phox^, and IL-6 protein expression levels in liver tissue by WB. (**G**) Correlation between IL-6 and p47^phox^ protein expression in liver tissue. (**H**–**L**) NOX4, gp91^phox^, p47^phox^, and IL-6 protein expression levels in skeletal muscle tissue by WB. (**M**) Correlation between IL-6 and p47^phox^ protein expression in skeletal muscle tissue. The molding effect is represented by *, * *p* < 0.05. The exercise effect is denoted by a, ^a^ *p* < 0.05, ^aa^ *p* < 0.01. The effect of combined exercise and drug intervention was indicated by c, ^c^ *p* < 0.05 and ^cc^ *p* < 0.01.

The liver pathology of ALD mice mainly showed hepatocyte steatosis and inflammatory damage. In the ALD group, the cell morphology was chaotic, the structure was incomplete, and there were a lot of red aggregations of inflammatory factors, and a large number of lipid droplets were accumulated in the hepatocytes. After exercise intervention, the degree of steatosis and inflammatory infiltration of the liver cells in mice were improved, and the cytoplasm of the liver cells was compact. The improvement was better when exercise and apocynin were combined, but inflammatory cell infiltration still existed (*p* < 0.05, *p* < 0.01, Figure 3A). In liver tissue, the expression of IL-6 protein in the ALD group was significantly higher than in the Con group (*p* < 0.05, Figure 3B,F), and the expression of p47^phox^ protein in the ALD + NOXI group and IL-6 protein in the ALD group were higher than in the ALD + Ex + NOXI group (*p* < 0.05, Figure 3B,E,F). There was a positive correlation between IL-6 and p47^phox^ in liver tissue, with a correlation coefficient of 0.7913, indicating a strong correlation between IL-6 and p47^phox^ (Figure 3G). In gastrocnemius muscle tissue, compared with the ALD group, the expression of p47^phox^ protein was increased in the ALD + NOXI group (*p* < 0.05, Figure 3H,K), and the expression of IL-6 protein was decreased in the ALD + Ex + NOXI group (*p* < 0.01, Figure 3H,L). The level of p47^phox^ protein in the ALD + Ex + NOXI group was significantly lower than that in the ALD + NOXI group (*p* < 0.01, Figure 3H,K). There was a low correlation between IL-6 and p47^phox^ protein in muscle tissue, with a correlation coefficient of 0.3000 (Figure 3M).

### 3.4. Drinking Alcohol during Exercise Aggravates Dyslipidemia, Which Had No Significant Effect after Apocynin Intervention

**Figure 4:**
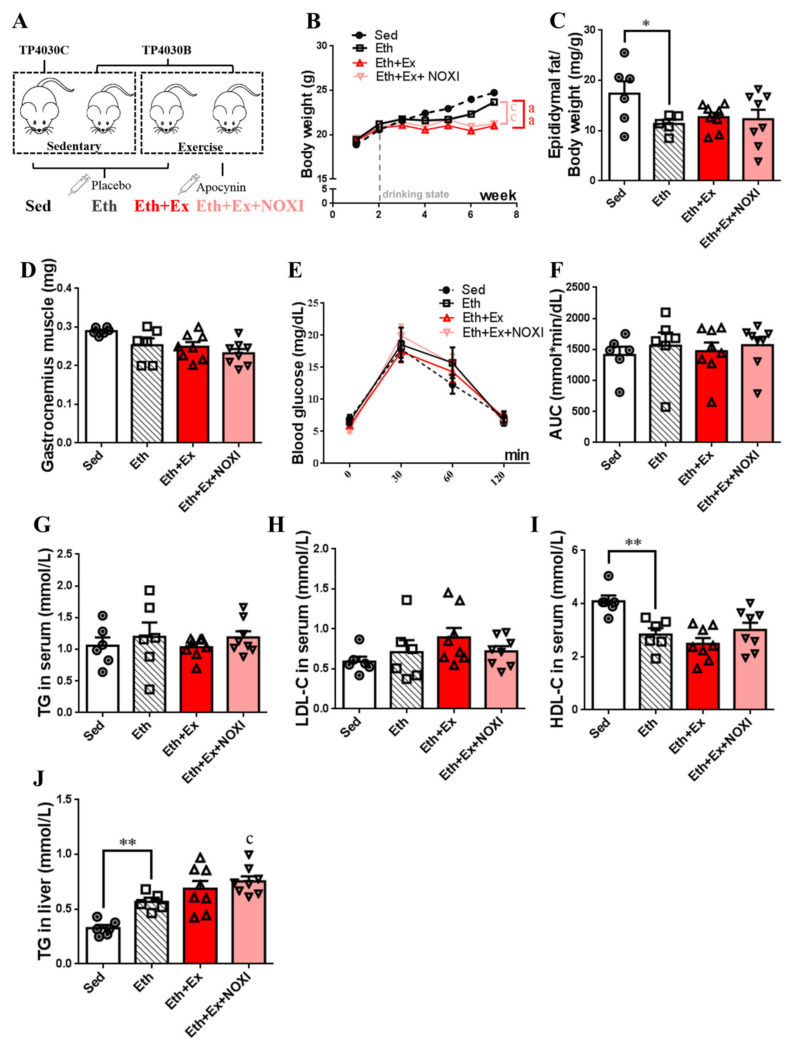
Drinking alcohol during exercise aggravates dyslipidemia, which had no significant effect after apocynin intervention. (**A**) Grouping of experimental animals. (**B**) Changes in body weight over 7 weeks. (**C**) Ratio of epididymal fat to body weight. (**D**) Gastrocnemius muscle weight. (**E**,**F**) Glucose change and area-under-the-curve in glucose tolerance test. (**G**,**J**) TG content in serum and liver tissue. (**H**) The level of LDL-C in serum. (**I**) The level of HDL-C in serum. (**A**–**J**) Sed group, *n* = 6; Eth group, *n* = 6; Eth + Ex group, *n* = 8; Eth + Ex + NOXI, *n* = 8. The molding effect is represented by *, * *p* < 0.05 and ** *p* < 0.01. The exercise effect is denoted by a, ^aa^ *p* < 0.01. The effect of combined exercise and drug intervention is indicated by c, ^c^ *p* < 0.05 and ^cc^ *p* < 0.01.

The changes in the main physiological indexes and lipid metabolism levels in mice induced by exercise under the drinking state are shown in Figure 4. Exercise intervention was performed for 6 weeks after 1 week of adaptive feeding, and alcohol and apocynin interventions were initiated simultaneously at week 3 of the trial (Figure 4A). The Eth + Ex group and Eth + Ex + NOXI group exhibited more significant weight loss than the Eth group (*p* < 0.01, Figure 4B). The epididymal relative fat content of the Eth group decreased significantly compared with the Sed group (*p* < 0.05, Figure 4C). Compared with the Sed group, the level of HDL-C in the Eth group was decreased significantly (*p* < 0.01, Figure 4I). In liver tissue, the level of TG in the Eth group was increased significantly compared with the Sed group (*p* < 0.01), and the Eth + Ex + NOXI group was higher in TG than the Eth group (*p* < 0.05, Figure 4J).

### 3.5. Drinking Alcohol during Exercise can Increase the Level of Inflammation and the Oxidative Stress in Tissues

**Figure 5:**
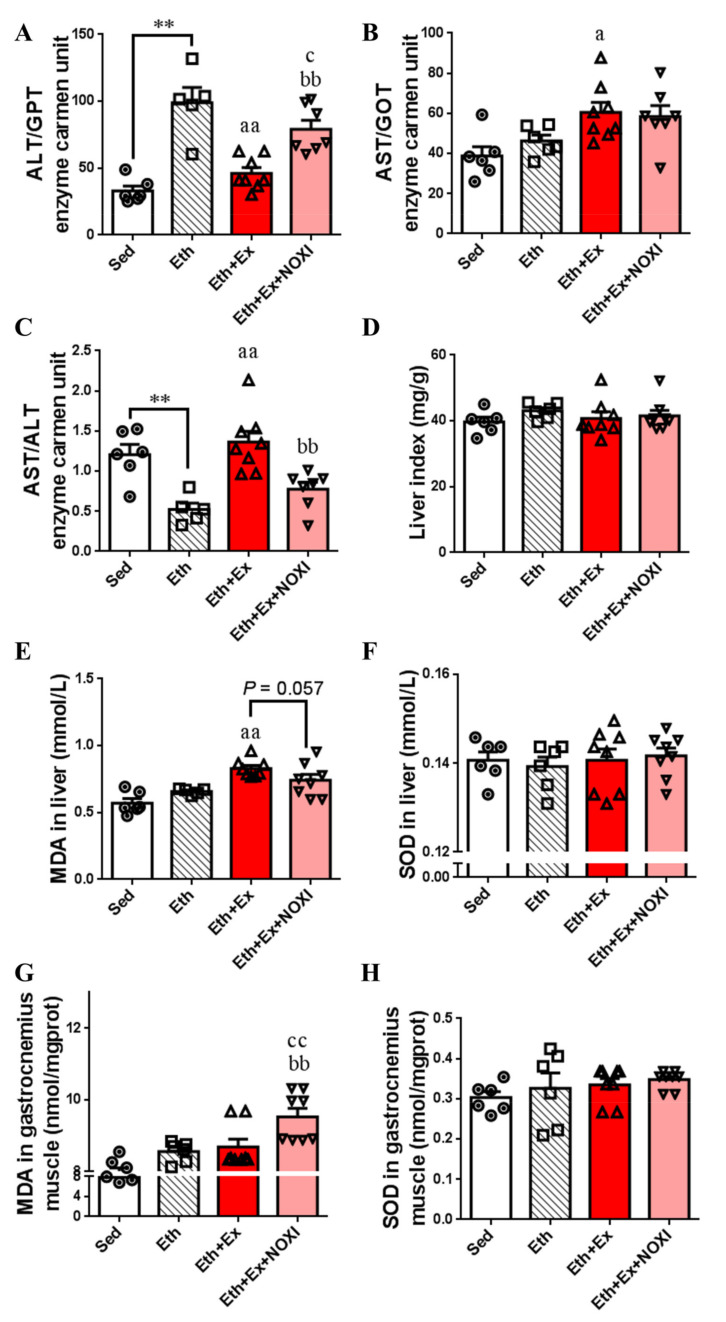
Exercise during drinking can increase levels of inflammation and aggravate the oxidative response of liver and skeletal muscle tissues. (**A**) The level of ALT in serum. (**B**) The level of AST in serum. (**C**) Serum AST/ALT values. (**D**) The level index. (**E**,**F**) MDA and SOD levels in liver tissue. (**G**,**H**) MDA and SOD levels in gastrocnemius muscle tissue. (**A**–**H**) The molding effect is represented by *, ** *p* < 0.01. The exercise effect is denoted by a, ^a^ *p* < 0.05 and ^aa^ *p* < 0.01. The drug effect is denoted by b, ^bb^ *p* < 0.01. The effect of combined exercise and drug intervention is indicated by c, ^c^ *p* < 0.05 and ^cc^ *p* < 0.01.

The influence of exercise on liver injury and the oxidative stress levels of the mice under the drinking state is shown in Figure 5. The level of ALT in the Eth group was higher than the Sed group (*p* < 0.01). Compared with the Eth group, the ALT level was decreased significantly in the Eth + Ex and Eth + Ex + NOXI groups. (*p* < 0.01, *p* < 0.05). The level of ALT was increased significantly after apocynin intervention (*p* < 0.01, Figure 5A). The level of AST in the Eth + Ex group was higher than that in the Eth group (*p* < 0.05, Figure 5B). The ALT levels in Eth group were lower than in the Sed group, and the Eth + Ex group was higher in ALT levels than the Eth group (*p* < 0.01). ALT levels were increased significantly after apocynin intervention (*p* < 0.05, Figure 5C). Compared with the Eth group, MDA levels in both liver tissue of the Eth + Ex group and the gastrocnemius muscle tissue of the Eth + Ex + NOXI group were significantly increased (*p* < 0.01, Figure 5E,G). Compared with the Eth + Ex group, after apocynin intervention, the levels of MDA in liver tissue decreased (*p* = 0.057, Figure 5E), and in gastrocnemius muscle tissue they increased significantly (*p* < 0.01, Figure 5G).

### 3.6. Exercise-Mediated Protein Expression of IL-6 and p47^phox^ in Liver Tissue

**Figure 6:**
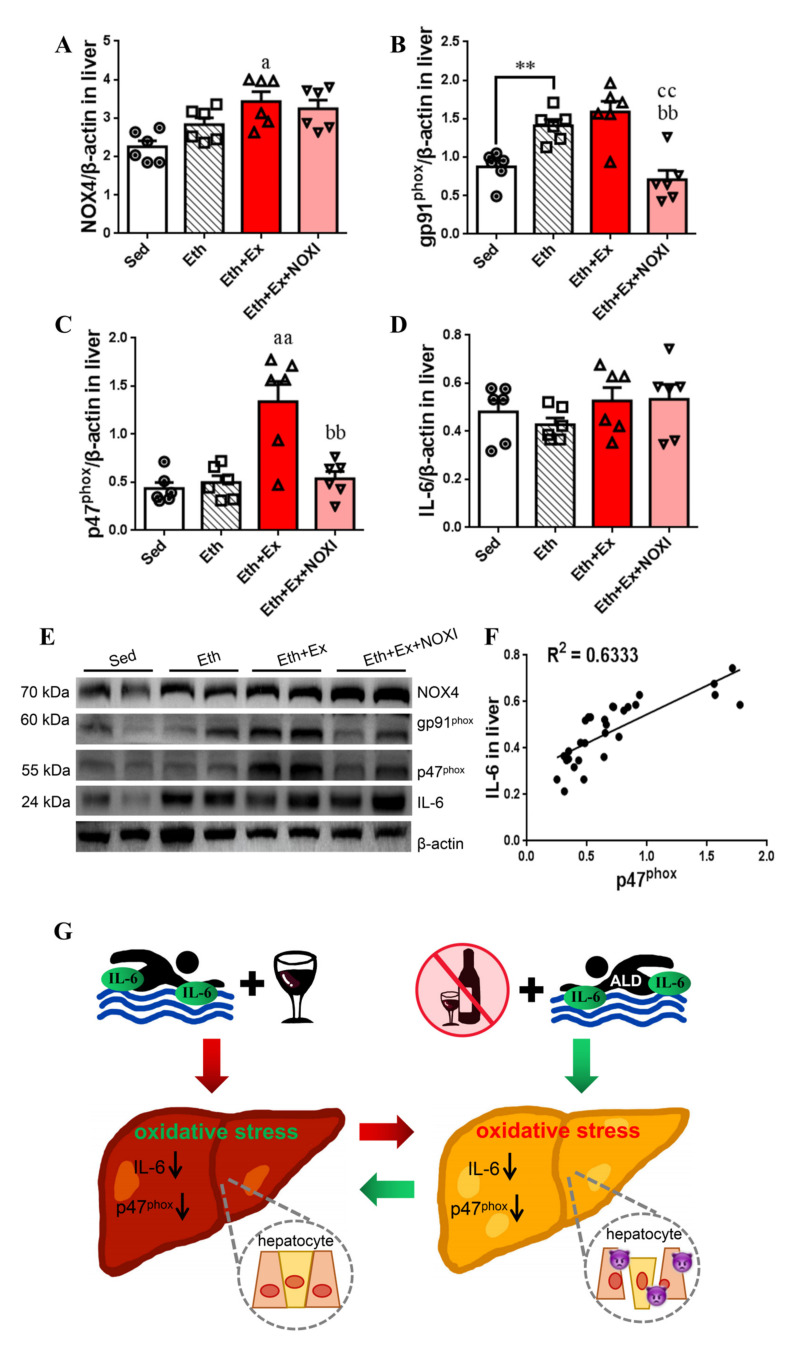
Verification of the correlation between IL-6 and p47^phox^ protein in liver tissue. (**A**–**E**) NOX4, gp91^phox^, p47^phox^, and IL-6 protein expression levels in liver tissue by WB. (**F**) Correlation between IL-6 and p47^phox^ protein expression in liver tissue. (**G**) Schematic diagram of effects of drinking alcohol during exercise and exercise intervention during the recovery phase of ALD on liver oxidative stress response and liver-cell inflammation damage. The molding effect is represented by *, ** *p* < 0.01. The exercise effect is denoted by a, ^a^ *p* < 0.05 and ^aa^ *p* < 0.01. The drug effect is denoted by b, ^bb^ *p* < 0.01. The effect of combined exercise and drug intervention is indicated by c, ^cc^ *p* < 0.01.

The influence of exercise on protein expression levels in liver tissue in mice under the drinking condition and the correlation between IL-6 and p47^phox^ are shown in Figure 6. The expression of gp91^phox^ protein in Eth group was higher than the Sed group (*p* < 0.01, Figure 6B,E). Compared with the Eth group, the expression of NOX4 protein was increased in the Eth + Ex group (*p* < 0.05, Figure 6A,E), gp91^phox^ protein was decreased significantly in the Eth + Ex + NOXI group (*p* < 0.01, Figure 6B,E), and p47^phox^ protein was increased significantly in the Eth + Ex group (*p* < 0.01, Figure 6C,E). Compared with the Eth + Ex group, gp91^phox^ and p47^phox^ protein expression decreased significantly after apocynin intervention (*p*< 0.01, Figure 6B,C). The dispersion degree of IL-6 protein expression in liver tissue was large after exercise intervention, and there was no significant difference among all groups (Figure 6D,E). IL-6 was positively correlated with p47^phox^ protein in liver tissue, and the correlation coefficient was 0.6333 (Figure 6F). It can be observed through the two experiments in this study that we found that exercise after abstinence can ameliorate hepatocyte damage and dyslipidemia, promote liver glycogen decomposition, and reduce liver function injury and fat accumulation. However, drinking alcohol during exercise can influence normal lipid metabolism and increase oxidative inflammation of the liver tissue. Exercise intervention significantly reduced p47^phox^ protein levels in liver tissue, and it improved ALD through the IL-6–p47^phox^ oxidative–stress axis (Figure 6G).

## 4. Discussion

TheLiber–DeCarli feeding model was closest to the pathway of alcohol intake and digestion after drinking, which could objectively reflect the normal metabolism of alcohol in the body [25,26]. In our experiment, we observed that compared with the Con and Sed groups, the alcohol-drinking mice were shown to be more irritable, react more slowly, exhibited poor appetite, lethargy, poor balance, and slow weight gain. In addition, mice in the ALD and Eth groups presented similar alcoholic steatosis and inflammatory pathologies, as reported in the study [27,28]. Although the ALD and ALD + NOXI groups underwent 6 weeks of self-recovery and drug therapy at the later stages of modeling, they were still unable to rapidly process glucose in the blood, and the structures of the hepatocytes were swollen and overlapping, and the hepatocyte cells were filled with fat vacuoles. TG levels in serum and liver tissue increased, and the levels of LDL-C and HDL-C also changed to varying degrees. Our results showed that liver injury was still in the early stage of ALD after 6 weeks of natural reversal and apocynin intervention, and the lipid metabolism of the mice was abnormal after alcohol intervention.

The content of ALT in liver tissue is about 100 times that in the serum. AST is mainly distributed in the myocardium, followed by the liver, skeletal muscle, and kidney, and its sensitivity is slightly weaker than that of ALT [29,30]. When the liver is in a pathological state, swelling and necrosis of the hepatocytes, aggravated inflammation, and other factors., result in increased permeability of the cell membranes, leakage of ALT into the cytoplasm and leakage of the AST in mitochondria into the blood, and increased content of both enzymes in the serum [31]. Mild liver injury is mainly caused by elevated ALT. AST increases only when liver cells are seriously damaged and pathological changes appear in the mitochondria [32]. Therefore, if AST/ALT decreases, it is mainly seen in the early stage of acute hepatitis or mild chronic hepatitis; when the hepatocyte damage is serious, AST and ALT significantly increased, and the higher the ratio is, the more serious the hepatocyte damage is. In this article, the AST and ALT values in the ALD + Ex + NOXI group were lower than in the ALD and ALD + NOXI groups (*p* < 0.05, *p* < 0.01), which showed that the liver function of mice recovered better under the combined intervention of exercise and apocynin. Surprisingly, drinking alcohol during exercise had little effect on lipid metabolism and aggravated liver injury (*p* < 0.01). The results of experiment 2 are the opposite of experiment 1 in these regards.

Jakob G Knudsen et al. fed mice with a high-fat diet which changed the regulating energy pathway; through 16 weeks of treadmill exercise training, the abnormal expression of the hepatocyte cytochrome P450 enzyme system was reversed, and the metabolic state of the liver was regulated [33]. Compared with the ALD and ALD + NOXI groups, the mice after exercise showed blood glucose rises that occurred more slowly and returned to the normal state more quickly, and the liver metabolism of the ALD mice was regulated and the lipid metabolism disorder was significantly improved. In experiment 2, drinking alcohol during exercise also produced similar changes in blood glucose as those observed in experiment 1. Compared with the Eth group, the Eth + Ex group increased lipid metabolism disorders, which were alleviated by the apocynin intervention during exercise.

This paper mainly observed the effect of exercise intervention on an ALD mouse model. Measuring MDA and SOD levels in liver tissue and gastrocnemius muscle tissue, respectively, which can indirectly reflect the body’s ability for free radical scavenging and its levels of antioxidants [34,35]. The experimental results showed that MDA levels in the gastrocnemius tissue increased in the exercise group because skeletal muscle is the effective organ of exercise, and positive changes inevitably occur [36,37]. Mauro Robson Torres de Castro et al. suggested that physical activity can change the oxidative inflammatory state of the liver, and exercise can reduce the expression of IL-6 in the liver [38]. The results of our paper confirm this conclusion. In liver tissue, SOD levels in the ALD + Ex + NOXI group were lower than the ALD + NOXI group (*p* < 0.01), and MDA levels in the Eth + Ex group were higher than the Eth and Eth + Ex + NOXI groups (*p* < 0.05). It was confirmed that exercise or exercise combined with apocynin could reduce liver tissue peroxidation damage during the recovery period of ALD and can interfere with the body’s ability to resist free radicals during alcohol consumption, which is similar to the conclusion obtained by Shen F [39].

Apocynin suppressed NOX4 and gp91^phox^ in normal mouse liver tissues, but this was not observed for p47^phox^ [13], and apocynin was more effective in NOX inhibition in ALD modeling [40,41,42]. Our research group reviewed the role of IL-6 in alcoholic liver disease and determined the relationship between IL-6 and p47^phox^ [43]. It is suggested that the mechanism of exercise-induced improvement of alcoholic liver disease is mediated by the IL-6–p47^phox^ oxidative-stress pathway. In this article, we tested our hypothesis with two experiments. In experiment 1, we changed the diet and activity levels of mice after the ALD model had been established to explore the effects of exercise and drugs on glucose and lipid metabolism, oxidative stress, and inflammatory injury in the recovery process of alcoholic liver disease. Western blot results showed that exercise intervention significantly downregulated the expression of IL-6 and p47^phox^ proteins in tissue, and the correlation was significantly higher in liver tissue than in gastrocnemius muscle tissue, with a correlation coefficient of 0.7913. Subsequently, we carried out experiment 2, which confirmed that exercise could regulate the expression of IL-6 and p47^phox^ in liver tissue through drinking alcohol during exercise. Western blot results were similar to experiment 1. Under the condition of drinking alcohol during exercise, there was a significantly positive correlation with p47^phox^ and IL-6 in liver tissue, and the correlation coefficient is 0.6333. Therefore, we believe that the internal molecular mechanism of long-term aerobic exercise in improving chronic inflammation in ALD is the IL-6–p47^phox^ axis.

## 5. Conclusions

Exercise, as a stress response, stimulates temporary inflammation and then puts the body in a state of long-term anti-inflammatory adaptation [44,45]. In the process of exercise stress, skeletal muscle is no longer regarded as a simple motor organ but also possesses endocrine function. IL-6 produced and released by skeletal muscle circulates to the corresponding target organs (such as the liver) through the blood, thereby improving immune function, maintaining central excitability, regulating enzyme activity, and improving the sensitivity of inflammatory mediators and other biological functions [46]. In this article, ALD mice were used as research objects to explore the effect of exercise regulation on the effects of the IL-6–p47^phox^ oxidative–stress axis on hepatocyte inflammatory damage. The conclusions were as follows: (1) 6 weeks of exercise intervention during the recovery phase of ALD may ameliorate hepatocyte damage and dyslipidemia through the IL-6–p47^phox^ oxidative–stress axis, and administering apocynin in combination could optimize this effect; (2) Drinking alcohol during exercise exacerbates dyslipidemia, oxidative stress, and downregulated expression of the IL-6–p47^phox^ axis–related protein in hepatocytes.

## Data Availability

Not applicable.
